# The Importance of Non-Coding RNAs in Neurodegenerative Processes of Diabetes-Related Molecular Pathways

**DOI:** 10.3390/jcm10010009

**Published:** 2020-12-23

**Authors:** Joanna Jarosz-Popek, Marta Wolska, Aleksandra Gasecka, Pamela Czajka, Daniel Jakubik, Lucia Sharif, Taqwa Adem, Wei-Ling Liu, Dagmara Mirowska-Guzel, Marek Postula, Ceren Eyileten

**Affiliations:** 1 Centre for Preclinical Research and Technology, Department of Experimental and Clinical Pharmacology, Medical University of Warsaw, 02-091 Warsaw, Poland; jjarosz@wum.edu.pl (J.J.-P.); wolska.marta@op.pl (M.W.); czajka.pamela@gmail.com (P.C.); djakubik@wum.edu.pl (D.J.); luciamsharif@gmail.com (L.S.); taqwa.adem@gmail.com (T.A.); liujenny.lj@gmail.com (W.-L.L.); dmirowska@wum.edu.pl (D.M.-G.); mpostula@wum.edu.pl (M.P.); 2 1st Chair and Department of Cardiology, Medical University of Warsaw, 02-091 Warsaw, Poland; aleksandra.gasecka@wum.edu.pl

**Keywords:** non-coding RNA, lncRNA, miRNA, miR, novel biomarker, treatment, biomarker

## Abstract

Diabetes mellitus (DM) is a complex condition and serious health problem, with growing occurrence of DM-associated complications occurring globally. Persistent hyperglycemia is confirmed as promoting neurovascular dysfunction leading to irreversible endothelial cell dysfunction, increased neuronal cell apoptosis, oxidative stress and inflammation. These collaboratively and individually result in micro- and macroangiopathy as well as neuropathy demonstrated by progressive neuronal loss. Recently, major efforts have been pursued to select not only useful diagnostic and prognostic biomarkers, but also novel therapeutic approaches. Both microRNAs (miRNAs) and long non-coding RNAs (lncRNAs) belong to a class of non-coding RNAs identified in most of the body fluids i.e., peripheral blood, cerebrospinal fluid, brain tissue and neurons. Numerous miRNAs, lncRNAs and their target genes are able to modulate signaling pathways known to play a role in the pathophysiology of progressive neuronal dysfunction. Therefore, they pose as promising biomarkers and treatment for the vast majority of neurodegenerative disorders. This review provides an overall assessment of both miRNAs’ and lncRNAs’ utility in decelerating progressive nervous system impairment, including neurodegeneration in diabetic pathways.

## 1. Introduction

Diabetes mellitus (DM) is one of the most common chronic diseases worldwide [1]. In accordance with the latest edition of the International Diabetes Federation reports, currently almost half a billion people suffer from diabetes and by 2045 this count will reach 700 million [2]. DM is defined by persistent hyperglycemia and defective metabolism of carbohydrates caused by decreased secretion and increased resistance of insulin as a consequence of β-cells dysfunction [3]. Nearly half of patients present with poorly controlled diabetes which leads to a series of macro- and microvascular complications including cardiovascular disease (CVD), diabetic neuropathies such as retinopathy, and corneal neuropathy [4]. Although recent studies have shed light upon a correlation between DM and nervous system complications, i.e., neuropathy, neurovascular dysfunction, and neuroinflammation resulting from progressive neurodegeneration, particular underlying mechanisms are yet to be fully elucidated [5,6].

Type 1 diabetes mellitus (T1DM) is a chronic disease, in which the autoimmune system destroys the pancreatic beta cells responsible for insulin production [7]. Insulin possesses anabolic and anti-catabolic properties and maintains homeostasis of carbohydrate metabolism. Meanwhile, insulin deficiency leads to constant hyperglycemia [8]. The role of immune breakdown, including the expansion of CD4+ and CD8+ autoreactive T cells as well as B lymphocytes responsible for the production of autoantibodies, is underlined in the pathogenesis of T1DM [9]. Many scientists have researched the correlation of T1DM with retinal neurodegeneration, one of the earliest complications in T1DM [10,11].

Type 2 diabetes (T2DM) is a potentially reversible disease, characterized by high blood glucose, insulin resistance and relative lack of insulin. Insulin resistance is the earliest abnormality and major pathophysiological factor of T2DM. The role of insulin signaling defects, glucose transportation defects or lipo-toxicity are underlined in a study of insulin resistance pathophysiology [12]. Another key component of T2DM onset and progression is B-cells apoptosis. Although genetic abnormalities also play a role in the mechanism of T2DM, an unhealthy diet as well as a sedentary lifestyle lead to obesity and are crucial factors in the disease’s pathophysiology [3]. Various reports indicate that there is a link between T2DM and the development of neurodegenerative diseases as well as exacerbation of the neurodegenerative processes [13].

The alteration of microRNA (miRNA, miR) and long non-coding RNA (lncRNA) expression along with their relation to the pathophysiological mechanisms of chronic hyperglycemia was documented in diabetic neuropathy. LncRNA are a class of RNA non-protein transcripts longer than 200 nucleotides, serving as transcriptional regulators able to impact cellular processes, i.e., proliferation, differentiation or apoptosis [14,15,16,17,18]. They participate in numerous biological processes such as regulation of gene expression through mRNA splicing, transcription regulation, translation regulation and genomic imprinting [14,15,16,17,18,19]. LncRNAs are capable of modifying cellular responses through down- or up-regulation in microvascular degeneration and in high glucose-induced neuronal injury. This should be taken into consideration when considering these proteins as a novel therapeutic approach in diabetic-induced neurodegeneration, along with miRNAs (other promising molecules also broadly analyzed) [20,21]. MiRNAs are small (18–25 nucleotides), endogenous, single-stranded and non-coding RNAs, that are able to modulate approximately 60% of mammalian protein coding genes post-transcriptionally. MiRNAs are considered to play a pivotal role in many common disorders, e.g., DM, CVD as well as ischemic stroke [16,18]. Moreover, particular miRNAs were found to upregulate or downregulate particular cellular responses in diabetes-induced neurovascular injuries. Therefore, it is hypothesized that certain miRNAs and lncRNAs could be novel biomarkers and could direct a novel therapeutic approach in diabetic neuropathies [22]. This review aims to provide an overall overview of the current knowledge of miRNAs and lncRNAs in neurodegeneration and neuro-regenerative processes resulting from DM.

## 2. MiRNAs and Their Link to Neurodegenerative Changes in Metabolic Pathways Related to Diabetes

A DM-induced persistent state of hyperglycemia is suggested to be a major cause of a variety of pathological pathways, including oxidative stress, apoptosis, inflammation and neurodegeneration. Oxidative stress is associated with inflammation and neurodegeneration due to formation and augmented concentration of reactive oxygen species (ROS) [23]. Hyperglycemia increases the production of ROS which results in a dysfunction in neuronal cells. Additionally, oxidative stress enhances an imbalance between endogenous ROS and antioxidant defense systems, initiating chronic inflammation and tissue damage [24]. MiRNAs regulate many biological processes, and are correlated with different aspects of complex diseases. Differential expressions of miRNAs were observed in patients with neurodegenerative diseases as well as DM, as they play an important role in regulating diabetes-induced inflammatory and neurodegenerative responses [25].

### 2.1. MicroRNAs Involved in Neurodegeneration and Regeneration

#### 2.1.1. MiRNAs in Diabetic Neuropathies

Diabetic peripheral neuropathy (DPN) is one of the most frequent persistent complications of all stages of DM, resulting in gradually spreading peripheral nerve damage. It is speculated that in a few years over 50 million diabetes patients worldwide will develop DPN [26]. DPN demonstrates symmetric, spreading proximally and mainly sensory progressive axonal loss as a result of chronic hyperglycemia and microangiopathy. Amongst the pathophysiological processes underlying DPN, increased oxidative stress, apoptosis ratio, mitochondrial dysfunction, chronic inflammation and accumulation of advanced glycation end products (AGEs) are deemed crucial. However, particular mechanisms have still not been fully elucidated [27,28]. Moreover, increasing evidence suggests that diabetic endothelial dysfunction is an early manifestation of DPN. Both DPN and diabetic endothelial dysfunction show similarities in induction of shared signaling pathways and seem to force each other [29,30]. Undoubtedly, diabetic endothelial dysfunction is a major cause of diminished neuronal perfusion resulting in reduced axonal reflexes, vasodilation and therefore, may promote neurodegeneration. Moreover, endothelial dysfunction is deemed crucial in the onset and progression of cerebral small vessel disease (CSVD), which may result in stroke and cognitive decline. Phosphodiesterase 3 (PDE3) is an enzyme detected in brain arteries which is known to play a key role in regulating endothelial function [31]. PDE3-targeting miR-221/miR-222 and miR-27a/miR-27b/miR-128 were identified in silico analysis. Overexpression of miR-27a-3p and miR-222-3p decreased the protein level of PDE3A in the in vitro model. MiR-221/miR-222 and miR-27a/miR-27b/miR-128 family impact pathways involved in immune modulation as well as cerebrovascular integrity and function. Targeting PDE3A by particular endothelial miRNAs may present as a suitable treatment for CSVD due to the capacity of these miRNAs to simultaneously affect various pathways related to CSVD and should consequently be studied more extensively in diabetic models [32].

One of the crucial factors involved in neuropathological processes is the small acidic polypeptide thymosin β4 (Tβ4) which promotes neuro-regeneration and reduces inflammation in diabetes-induced injury. Alongside this, miRNAs are known to influence some pathways involved in the onset and progression of neuronal dysfunction. Therefore, Wang et al. [33] analyzed the neuroprotective effects of Tβ4 in DPN on miR-146a both in vivo and in vitro. It was found that the Tβ4 injection reversed the inhibitory effect of diabetes by miR-146a upregulation. Increased miR-146a expression ameliorated the motor and sensory function of nerves, and improved nerve fiber density and regional blood flow in animal models, whereas endogenous inhibition of miR-146a reversed the positive effect of Tβ4 on endothelial cells. High glucose levels caused downregulation of miR-146a and upregulation of its target genes *IRAK1*, *TRAF6* and *p-NFkB* further causing increased pro-inflammatory mediators, such as MCP-1 and VCAM-1 levels. Taken together, the study showed that Tβ4 promoted the neuroprotective role via upregulation of miR-146a in diabetic subjects by the inhibition of inflammatory mediators [34]. Furthermore, the same research team aimed to analyze the effect of sildenafil on miRNA expression in distal axons of embryonic cortical neurons. Sildenafil is suggested to upregulate the expression of miR-146a in dorsal root ganglia (DRG) and ameliorates neuropathy [33,35]. In high glucose conditions, axonal miR-146a expression was significantly reduced. This resulted in a significant increase in target genes *IRAK1* and *TRAF6*. MiR-146a treatment on DRG neurons diminished axonal mRNA and protein levels of IRAK1 and TRAF6 (proinflammatory mediators) forcing axonal lengthening. In line with these findings, sildenafil use reversed high glucose-induced axonal injury by downregulation of miR-146a. Taken together, miR-146a seems to serve as a novel therapeutic approach in high glucose-induced neuropathy [36].

**Figure 1:**
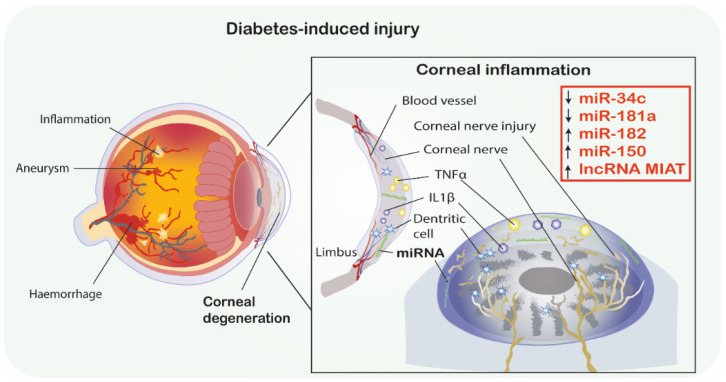
Diabetic corneal neuropathy. MiRNAs as promising therapeutics in diabetes-induced corneal degeneration. ↑ indicates the mimic-use as treatment, ↓ indicates inhibitor-use as treatment. Abbreviations: IL-1β, Interleukin 1β; TNF-α, Tumor necrosis factor α; MIAT, Myocardial infarction associated transcript; miRNA-miR, microRNA; lncRNA, long non-coding RNA.

Diabetic corneal neuropathy (DCN) belongs to a variety of common diabetic-associated ophthalmic complications. DCN is demonstrated by simultaneously decreased neuronal fiber density and length, resulting in the onset of neurotrophic ulcer and progressive visual loss [37]. In this regard, Hu et al. conducted a study to analyze the role of miR-34c in DCN associated with T1DM. Researchers utilized both in vivo and in vitro studies. In trigeminal tissue in the diabetic mouse model, miR-34c expression was significantly increased compared to controls. The in vitro studies showed that inhibiting miR-34c resulted in an increased growth of neurites and increased total length of trigeminal sensory neurons. The in vivo study showed that subconjunctival injections of miR-34c antagomir (inhibitor of miRNA) increased corneal nerve density and promoted epithelial wound healing via increased nerve fiber regeneration. In terms of the underlying pathological mechanisms, the autophagy-related proteins, namely, Atg4B and LC3-II, which promote autophagy, were downregulated in diabetic mice trigeminal ganglia. This study predicted the possible interaction of miR-34a and Atg4B by using an in silico tool and confirmed it also with an in vitro experimental analysis. Thus, this study concluded that miR-34c silencing can promote neuroprotective effects in nerve injury via the upregulation of Atg4B and autophagy promotion. Ultimately, the silencing of this miRNA may present as a promising approach by enhancing corneal nerve regeneration and epithelial wound healing in diabetic corneal neuropathies [38] (Figure 1).

Moreover, the same research group investigated the neuroprotective role of miR-181a in mice trigeminal ganglia neurons serving as a model of the T1DM corneal nerve. Firstly, it was found that miR-181a is upregulated in diabetic trigeminal cells. The in vitro study showed that trigeminal cells (cultured in a high glucose environment and treated with miR-181a antagomir) showed a significant increase in axonal growth compared to the control groups. Moreover, subconjunctival injections of the miR-181a inhibitor, performed after epithelial scraping, accelerated the corneal epithelium damage repair in vivo. The density of the corneal plexus in the treated group was higher than in negative-controls. Furthermore, miR-181a antagomir therapy was correlated with increased expression of ATG5, LC3B-II and Bcl-2 proteins which are associated with autophagy promotion and apoptosis inhibition. In summary, the miR-181a antagomir treatment was correlated with the upregulation of anti-apoptotic and autophagy enhancing proteins as well as acceleration of neuronal axon growth and corneal tissue damage repair in diabetic mice. Thus, miR-181a inhibition seems to have a neuroprotective effect on diabetic corneal nerve [39] (Figure 1).

An important gene involved in neuroprotection, especially sensory neurons in diabetics, is *SIRT1*. Wang et al. [33] conducted a study to analyze the correlation between corneal tissue regeneration and miRNA associated with *SIRT1* in diabetic mice with corneal neuropathy. The study reported that *SIRT1* was downregulated in the trigeminal sensory neurons of diabetic mice. Additionally, *SIRT1* was overexpressed via subconjunctival injection and in miRNA microarray and PCR validation analysis in trigeminal cells. Ultimately, the study showed that miR-182 was significantly upregulated by *SIRT1* in these cells. Importantly, the overexpression of miR-182 promoted axonal growth in diabetic trigeminal cells and therefore reversed the negative impact of high glucose conditions. The in vivo investigations showed that miR-182 injections increased corneal nerve density, improved corneal sensation and reduced corneal epithelium defect. The in silico analysis showed that *NOX4*, associated with ROS production, is increased in diabetic trigeminal cells, and is additionally a direct target gene of miR-182. The miR-182 injection caused a downregulation of *NOX4*, thus promoting corneal nerve regeneration in diabetic mice. In conclusion, the research presented a correlation between *SIRT1*, miR-182 and *NOX4* as well as their interactions regarding corneal healing and trigeminal nerve innervation in diabetes [40].

Wu et al. [41] analyzed retinal miRNA expression in diabetic retinopathies utilizing a STZ (streptozocin)-induced diabetic animal model. STZ injections caused retinal capillary dilatation, interstitial edema and various other pathological changes of the capillary basement membrane, endothelial cells and mitochondria. MiRNA microarray analysis showed that 37 retinal miRNAs were altered in diabetic rats. However, only 17 of these were confirmed in qRT-PCR analysis. It was found that miR-182, miR-96, miR-183, miR-211, miR-204 and miR-124 were increased with diabetic retinopathy development, whereas miR-199a-3p, miR-10b, miR-10a, miR-219-2-3p, miR-144 and miR-338 were decreased. Importantly, authors mentioned that important angiogenesis factors, including VEGF and PEDF, are direct targets of miR-199a-3p and miR-363, suggesting that these miRNAs could be crucial for capillary changes in diabetic retinopathy. Thus, specific miRNAs are associated with DR development and their modulation may be a novel therapeutic target for DR treatment [41], (Table 1).

#### 2.1.2. MiRNAs Involved in Insulin Signaling Pathways in Neurodegeneration

Alzheimer’s disease (AD) is the most common neurodegenerative disorder demonstrated by β-amyloid and tau protein aggregation in the brain that results in gradual tissue atrophy, nerve loss and ultimately progressive cognitive impairment. The underlying mechanism of AD has been extensively researched [48]. One of the postulated hypotheses is the alteration of insulin signaling which has been linked to diabetes and neurodegenerative disease development [49]. The role of miR-302 in neuroprotection against neurotoxicity induced by amyloid-β (Aβ) has been studied both in vitro and in vivo. MiR-302 transfection protected against Aβ-induced apoptosis, mitochondrial dysfunction and insulin resistance via stimulating the PI3K/Akt signaling pathway. Additionally, miR-302 activated the Akt/GSK3β axis which attenuated tau hyperphosphorylation involved in AD pathogenesis. Besides, treatment of miR-302 inhibits ROS accumulation through Akt-induced upregulation of Nrf2/HO-1 pathway and therefore protects against Aβ-induced neurotoxicity. Nrf2 is particularly involved in protection against oxidative stress impairment via increasing antioxidant reaction agents such as HO-1 [50]. Further analysis showed that overexpression of miR-302 inhibited PTEN expression via Akt signaling pathway activation which induced Nanog expression. Of note, reduced Nanog expression was found in AD patients. Silencing of Nanog expression was associated with tau hyperphosphorylation and neurotoxicity due to Akt/GSK3 axis inhibition [51]. MiR-302 treatment re-activated Akt/GSK3 signaling and inhibited AD progression. In addition, miR-302 is encoded in the *LARP7* gene and amongst patients with AD, the expression of *LARP7* was markedly decreased [44,52]. Overall, miR-302 is able to prevent AD progression through the activation of the Akt signaling pathway [44].

Studies have shown that the inhibition of *PDCD4* gene decreased apoptosis, and miR-21 was found as a direct target of *PDCD4* [53,54]. This explains why miR-21 treatment in neuronal damage due to diabetes was studied in DM rats with cerebral infarction (CI). Notably, the DM+CI rats which were transfected with miR-21 mimic and those that were transfected with PDCD4 siRNA (si-PDCD4) demonstrate better scores in motoric tests than in control groups. Additionally, analysis of rat brain tissue with miR-21 mimics DM+CI demonstrated decreased expression of proapoptotic markers, namely PTEN, FasL and PDCD4. Neuron differentiation markers (NeuN protein and neural-specific βIII-tubulin) were highly upregulated [55,56]. This evidence suggested that miR-21 may promote nerve cell regeneration via diminishing the apoptosis ratio by PDCD4 downregulation [45].

Venkat et al. [46] analyzed the role of miR-126 and exosomes derived from mouse brain endothelial cells (EC-exo) as a treatment for stroke in diabetic mice. In vivo analysis showed DM-stroke mice were injected with EC-exo or inhibitors of miR-126 + EC-exo. In the DM-stroke model, expression of miR-126 both in serum and brain tissue were significantly decreased when compared to mice with stroke without diabetes. Treatment with EC-exo significantly increased miR-126 expression in serum and brain tissue. Importantly, DM-stroke mice treated with EC-exo had an increased axon and myelin density, vascular density and arterial diameter when compared to control groups. EC-exo treatment was correlated with improvement of cognitive abilities and neurological function. Interestingly, this positive effect was not observed in case of miR-126 knockdown in EC-exo treatment, thus miR-126 seems to play a regulatory role in EC-exo neuro-regenerative effect. Similar findings were reported in vitro, as downregulation of miR-126 resulted in reduced angiogenesis and axon outgrowth. Thus, studies have suggested that miR-126 may play a key role in EC-exo neurorestorative effects in DM-stroke mice [46].

TRPM7 is responsible for magnesium homeostasis, therefore is also involved in glucose and insulin metabolism, whereas miR-34a plays a role in beta-cell apoptosis [57,58]. Zhang et al. [43] conducted a study to investigate the effect of silencing TRPM7/miR-34a in mice with T1DM. Firstly, both TRPM7 and miR-34a were found to be upregulated in T1DM mice. Silencing of TRPM7/miR-34a resulted in improved spatial learning and memory abilities. Moreover, an increased number of neurons in the hippocampal region and improved neuronal structures were observed after TRPM7/miR-34a silencing compared to control and T1DM groups. Reduction in T1DM typical hippocampal changes like swollen mitochondria, vacuolar degeneration and apoptosis symptoms were observed after TRPM7/miR-34a silencing. Apoptosis rate was reduced as the expressions of pro-apoptotic Bax, cyt-c, and cleaved-caspase-3 were significantly decreased, while Bcl-2, an important anti-apoptotic protein, increased notably. Consequently, TRPM7/miR-34a silencing can improve spatial cognitive function and hippocampal neurogenesis in T1DM mice [43]. Further clinical investigations are needed to evaluate the role of TRPM7/miR-34a in T1DM treatment (Table 1).

### 2.2. Dicer

Dicer is an endoribonuclease acting in the maturation of miRNA [59]. By cooperation with various proteins, Dicer processes miRNA precursors into mature, fully functional miRNAs [60]. The role of Dicer in pituitary dysfunction, neurodegeneration and development of obesity was determined by Schneeberger et al. [42]. Specific hypothalamic neurons (i.e., Agouti-related protein—AgRP, neuropeptide Y—NPY, pro-opiomelanocortin—POMC and cocaine and amphetamine-related transcript—CART) expressing orexigenic and anorexigenic neuropeptides involved in metabolism regulation were studied. Dicer expression was found in both AgRP and POMC neurons. An association between nutrient availability and expression of Dicer was found, as fasting was positively correlated with Dicer. Furthermore, Dicer deficiency in POMC neurons was established in an animal model. Dicer-deficient mice exhibited obesity as well as energy balance and glucose metabolism alterations. In addition, a deletion of Dicer in mice has caused an altered pituitary-adrenal axis, defined as a distinct deficiency of adrenocorticotropic hormone, with normal levels of other pituitary hormones as well as secondary hypoadrenalism. Lack of Dicer also resulted in strict neurodegeneration of POMC neurons, showing a 70% decrease in this subset of neurons in young mice. Taken together, Dicer deletion in POMC neuronal tissue leads to neurodegeneration as well as early onset of obesity and its following metabolic complications. As Dicer is a crucial enzyme for miRNAs formation, its deficiency causes inhibition of miRNA maturation, which leads to disruption of metabolic homeostasis regulated by miRNAs. As discussed above, many miRNAs play an important role in neuronal damage in DM, therefore Dicer alteration is indirectly associated with neurodegeneration/neurogenesis due to diabetes [42].

## 3. LncRNAs and Their Links to Neurogenesis and Nerve Regeneration in Diabetes

Recent evaluations strengthen the major role of lncRNAs in a variety of DM-induced disorders demonstrated by progressive nerve loss i.e., AD, Parkinson’s disease (PD), Huntington disease (HD) or IS. LncRNAs utility is broadly discussed with regard not only to diagnosis or prognosis but also as a therapeutic approach. Studies also suggest their crucial role in reversing these effects via promoting signaling pathways involved in nerve regeneration. Therefore, analysis of particular mechanisms involved in both neuronal injury and repairment seems crucial.

### 3.1. LncRNAs Involved in Neurodegeneration and Regeneration

#### 3.1.1. LncRNAs in Diabetic Retinopathy

**Figure 2:**
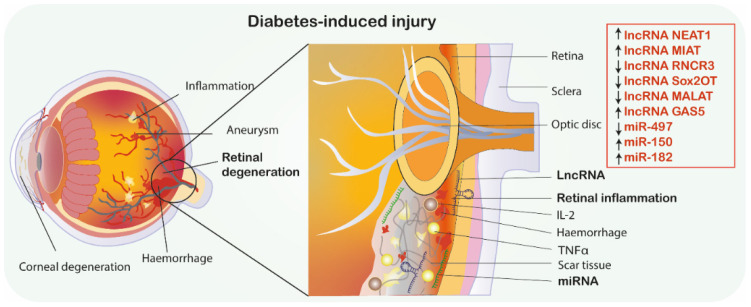
Diabetic retinopathy. MiRNAs/lncRNAs as promising therapeutics in diabetes-induced retinal degeneration. ↑ indicates the mimic-use as treatment, ↓ indicates inhibitor-use as treatment. Abbreviations: GAS5, Growth arrest-specific transcript 5; IL-2, Interleukin 2; MIAT, Myocardial infarction associated transcript; NEAT1, Nuclear paraspeckle assembly transcript 1; RNCR3, Retinal non-coding RNA3, Sox2OT, Sox2 overlapping transcript; TNF-α, Tumor necrosis factor α; MALAT1, metastasis-associated lung adenocarcinoma transcript 1; miRNA-miR, microRNA; lncRNA, long non-coding RNA.

Neurovascular dysfunction is a primary and major cause of diabetes complications, and nervous and vascular systems are regulated by mutual mediators. Studies showed that alteration of lncRNAs is involved in microvascular degeneration and high glucose-induced neuron injury [20,61,62]. Understanding underlying mechanisms responsible for neurovascular interactions could contribute to discovering novel therapeutic strategies. Therefore, Jiang et al. [19] analyzed the role of lncRNA myocardial infarction associated transcript (MIAT), which is found to be highly expressed in neurons and glial cells under hypoxia and oxidative stress conditions in diabetic retinopathy. In vitro evaluation revealed that MIAT inhibition significantly diminished gliosis via downregulation of both glial fibrillary acidic protein (GFAP) and vimentin (known as markers of Müller glial cells). Downregulation of those mediators by MIAT inhibition increased oxidative stress, apoptosis ratio, mitochondrial depolarization and reduced Müller cells viability. These results suggested that MIAT knockdown promoted neurovascular damage. MIAT knockdown significantly enhanced microvascular progressive damage. Further evaluation demonstrated that expression of neurovascular regulators, namely BDNF, NGF, NT3, Ang-1 and VEGF was downregulated by MIAT knockdown. Moreover, the study found that miR-150-5p can directly target MIAT and VEGF, suggesting its involvement in maintenance of neurovascular functionality. Hence, authors showed that lncRNA MIAT also plays an important role in microvascular dysfunction induced by DM and MIAT/miR-150/VEGF axis may represent a further pharmacological target for treating neurovascular-related disorders [19] (Figure 2, Table 2).

Growth factors modulate the physiological growth, formation and restoration of all tissues, including neuro-regeneration, and are also affected by pathological conditions [63,64]. VEGF is primarily responsible for functioning of endothelial cell proliferation and migration, as well as collagen production. Importantly, VEGF has been correlated with induced permeability of the blood–retina barrier and increased neovascularization in DM [65]. While VEGF is suggested as a promising biomarker of early stages of diabetic retinopathy, further correlation analysis should be conducted between lncRNAs and VEGF. Apart from VEGF, another important growth factor, which is involved in both neurodegenerative disease and diabetes, is BDNF [66,67,68,69]. It plays an important role in nervous system maturation while supporting the survival of neurons and neuro-regeneration after injuries. Many studies have shown the correlation of BDNF with miRNAs in diabetes-induced neurodegeneration. Studies are not only limited to in vitro and in vivo analysis, but several human studies were also conducted regarding BDNF-miRNAs interaction in diabetes, AD, PD, HD, multiple sclerosis and ischemic stroke [70]. On the other hand, very limited studies have shown the relation of lncRNAs with BDNF in neurodegenerative disease and diabetes, which are mainly in vitro and in vivo analysis [47,71,72,73,74]. For example, a previous study showed that high glucose condition caused Müller glial cells apoptosis by downregulating lncRNA NEAT1. NEAT1 treatment suppressed the apoptosis of retina Müller glial cells after diabetic retinopathy through modulating miR-497/BDNF cascade (by downregulating miR-497 and upregulating BDNF) [47]. Therefore, further studies are needed aiming to analyze the interaction between BDNF and lncRNAs in diabetes-induced neurodegeneration (Figure 2).

Another similar study aimed to analyze whether lncRNA RNCR3 mediate DM-induced retinal neurodegeneration by assessment of retinal glial reactivity which is an early manifestation of retinopathy. Both in vivo and in vitro evaluation revealed that RNCR3 knockdown lead to significant reduction of glial cell reactivity, demonstrated by diminished expression of GFAP, vimentin, proinflammatory cytokines such as IL-2, VEGF, MCP-1 or TNFα and decreased ratio of apoptotic retinal cells. Additionally, in vitro analysis revealed that RNCR3 knockdown can reverse progressive visual function loss. Thus, RNCR3 may be a promising target in preventing DM-related retinal neurodegeneration [75].

The role of lncRNA Sox2OT in diabetic retinopathy was also investigated. Previous study involved in vitro model of retinal ganglion cells (RGCs) and in vivo model of diabetic mice. Firstly, it was found that high glucose levels and oxidative stress downregulated Sox2OT expression in vitro. Similar findings were also reported in an animal model. Additionally, mice that were injected with a Sox2OT-inhibitor improved visual function, showing the protective role of Sox2OT inhibitor on RGC. Moreover, Sox2OT knockdown decreased ROS production and upregulated the activity of oxidative stress-related enzymes, which decreased oxidative stress-induced neuronal dysfunction both in in vitro and in vivo analysis. Furthermore, Sox2OT knockdown caused activation of NRF2/ARE signaling pathway. NRF2 is an important transactivator and activates ARE-dependent gene expression of antioxidative and cytoprotective proteins including HO-1 [80]. Taken together, Sox2OT knockdown protects against high glucose-induced RGC injury through the NRF2/HO1 signaling pathway, and seems to be a promising therapeutic approach for the prevention and treatment of diabetes-induced retinal neurodegeneration [77].

Another promising lncRNA MALAT1 on diabetic retinal neurodegeneration was studied by Zhang et al. [78], who found that retinal MALAT1 was upregulated in STZ-induced diabetic animal models. MALAT1 was silenced by MALAT1-siRNA injection in order to define its role in the retinal neurodegenerative process. Scotopic and photopic electroretinograms were performed to reflect the morphological and functional changes in rod and cone cells. The a-wave and b-wave amplitudes as well as outer nuclear layer thickness were lower both in diabetic control and diabetic MALAT1-siRNA models compared to normal control group. However, the amplitudes and outer nuclear layer were still significantly higher and thicker in the MALAT1-siRNA mice compared to diabetic control group, suggesting that silencing of MALAT1 shows protective effects on retinal photoreceptors and decreases retinal neurodegeneration due to diabetes [78] (Figure 2, Table 2).

LncRNA GAS5 has been documented to serve an important role in numerous signaling pathways, including insulin, mTOR and AKT [81,82,83]. Jiang et al. [79] investigated the influence of lncRNA GAS5 on endoplasmic reticulum stress and apoptosis in diabetic retinopathy. In this study, HG condition in retinal pigment epithelial cells resulted in increased endoplasmic reticulum stress, enhanced apoptosis and inflammation. The treatment of lncRNA GAS5 under HG conditions significantly reduced ER stress, apoptosis as well as inflammatory cytokines, such as TNF-α, IL-1β and IL-6. Consequently, the study indicates that lncRNA GAS5 plays a crucial role in diabetic retinopathy pathogenesis and further studies are needed to clarify its therapeutic potential [79].

#### 3.1.2. LncRNA Involved in Neuronal Apoptosis, Autophagy and Oxidative Stress

DM associated oxidative stress and apoptosis are strongly suggested as crucial factors in progressive neurodegeneration and play a role in promoting earlier onset of various diseases including dementia, AD. Accumulating evidence indicates a significant role of lncRNAs in inhibition of pathways involved in progressive neurogenesis, therefore their potential therapeutic usage is postulated. The study by Yu et al. [76] aimed to investigate both in vitro and in vivo lncRNA H19 roles in DM-induced oxidative stress and apoptosis in hippocampal neurons. LncRNA H19 is known as a crucial glucose metabolism factor and is associated with insulin-like growth factor 2 (IGF2), a regulator of oxidative stress and apoptosis via targeting Bax, caspase-3 and Bcl-2. Downregulation of lncRNA H19 suppressed IGF2 methylation, which in turn increased the expression of IGF2 and therefore inhibited apoptosis as well as oxidative stress in hippocampal neurons. Therefore, it was hypothesized that downregulation of lncRNA H19 could be a potential therapeutic target in diminishing neurodegeneration [76].

## 4. Concluding Remarks and Limitations

Increasing incidence of DM and its complications constitute a major medical care burden worldwide. Asymptomatic onset and constant progression of DM-associated neurovascular complications in line with limited diagnostic and therapeutic strategies are deemed crucial in searching for novel biomarkers and therapeutics. Since miRNAs and lncRNAs seem to play pivotal roles in modulation of DM-induced neurodegeneration, their molecular relation is broadly discussed. Despite the fact that underlying mechanisms of diabetic neurodegeneration are still not fully recognized, prior studies highlighted the pivotal role of apoptosis, oxidative stress, inflammation and mitochondrial dysfunction. High glucose-induced cell apoptosis and nerve degeneration along with a reduced rate of nerve regeneration play an important role in the progression of peripheral neuropathy among diabetic patients [84]. The correlation between non-coding RNAs (including miRNAs and lncRNAs) and nerve regeneration has been demonstrated in numerous studies. There are several non-coding RNAs found to be downregulated or upregulated in DM. Several studies demonstrated the promising role of these molecules as potential therapeutic approaches in miRNA- and lncRNA-based novel treatments. As described above, this novel treatment can be achieved by using antagonists or mimics of miRNAs/lncRNAs, as some of those molecules’ silencing shows the protective effect, whereas some of these show the protective effect by overexpressing. Yet the detailed mechanism of action of described miRNAs and lncRNAs on neurodegeneration due to diabetes-related oxidative stress and inflammation has not been fully explained and more studies need to be conducted.

## Figures and Tables

**Table 1:** Potential miRNAs involved in neurodegeneration.

Ref	Analyzed miRNAs and Their Deregulation	Analyzed mRNAs	Pathophysiological Mechanism and Axis	Methodology	Conclusion
[19]	↑miR-150	VEGF BDNF, NGF, NT-3, Ang-1	Oxidative stress, Apoptosis Hypoxia, Angiogenesis MIAT/miR-150-5p/VEGF	STZ-induced diabetes model *, in vitro, in vivo, Mice, cornea and retina * Study used single injection of STZ to mice, 60 mg/kg used to induce diabetes. Seven days after STZ injection, animals with blood glucose levels > 16.7 mmol/L were included in diabetic group.	MIAT regulates neural and vascular cell function via MIAT/miR-150-5p/VEGF network. MIAT knockdown leads to cerebral microvascular degeneration, progressive neuronal loss and neurodegeneration, behavioral deficits in a CNS neurovascular disorder, Alzheimer’s disease. MIAT may represent a pharmacological target for treating neurovascular-related disorders
[42]	No miRNA, Dicer	Nhlrc1, Park2, Rps24, Rps9UN Agrp, Cart, Pomc, Npy, Crh, Mc3r, Mc4r, Tpit, Crhr1, Ntrk2, Myc, Naglu, Acp2, UN Dicer UN Drosha, UN Dgcr8, UN Ago2, UN Pit-1, UN Gh, UN Tshβ,	Apoptosis, Inflammation, Autophagy	The role of Dicer in pituitary dysfunction and neurodegeneration, In vivo, In vitro, POMCDicerKO mice, pituitary gland, hypothalamus,	The absence of Dicer protein in pituitary neurons in mice leads to impaired neuronal function, development of obesity and neurodegenerative processes
[40]	↑miR-182	NOX4	Oxidative stress	T2DM model In silico, In vivo, BKS.Cg-m+/+Leprdb/J (db/db) mice as the model of T2DM, cornea	Sirt1 binds to miR-182 promoter and modulates its transcription. MiR-182 plays a key role in DM-induced corneal nerve regeneration via targeting NOX4. Administration of miR-182 lead to corneal healing effect in diabetic mice.
[43]	↑miR-34a	TRPM7 ICA IAA GAD-Ab Bax Cyt-c Caspase-3 Bcl-2	Apoptosis TRPM7/miR-34a	T1DM model STZ-induced T1DM, In vitro, In vivo, mice, hippocamp	Inhibition of TRPM7/miR-34a axis decreased apoptosis markers, improved spatial cognitive function and promoted hippocampal neurogenesis in mice with DM.
[32]	↑miR-27a-3p↑ miR-222-3p	PDE3A	CSVD	Endothelial dysfunction In silico, In vitro, hCMEC/D3 cell line model of cerebral endothelial micro-vessel cells	Increased levels of miR-222-3p and miR-27-3p diminished expression of PDE3A in cerebral endothelial cells via decreasing ischemic penumbra and diminishing damage caused by CSVD, therefore may play a role in endothelial function and improve cerebral circulation.
[33]	↓miR-146a	IRAK1, TRAF6 Caspase-3	Apoptosis, Inflammation	T2DM In vitro, In vivo, BKS.Cg-m+/+Leprdb/J (db/db) mice as the model of T2DM, DRGs neurons	DM increased IRAK1, TRAF6, caspase-3 and decreased miR-146a expression in DRG neurons. Sildenafil treatment reversed those effects. Mir-146a may play a crucial role in DM-induced DRG apoptosis.
[44]	↑ miR-302	Nrf2 HO-1 Nanog PTEN	Oxidative stress, Akt/Nfr2/HO-1 Apoptosis, Akt/GSK3, PI3K/Akt	AD and neurodegeneration in insulin signaling pathways, In silico, In vitro, In vivo, Human AD patients’ blood samples, human neuroblastoma SK-N-MC cells	Overexpression of miR-302 reduces Aβ-induced oxidative stress via activating Akt/Nfr2/HO-1, reduces mitochondrial dysfunction via Nrf2, reduces neurotoxicity and apoptosis via PI3K/Akt pathway. MiR-302 upregulation results with activating Akt/GSK2B by inhibition of PTEN and therefore targets Nanong which is a proliferation factor.MiR-302 can protect neuronal cells by restoring impaired insulin signaling via the prevention of Aβ-induced neurotoxicity. MiR-302 plays a neuroprotective role.
[45]	↑ miR-21	NeuN, GFAP, β-III-Tubulin, PDCD4 HNA, Nestine, PTEN, FasL, PDCD4	Apoptosis	Alloxan-induced diabetes *, In vitro, In vivo, Diabetic rats, brain frontal pole * Rats were intraperitoneally injected with 100 mg/kg alloxan for two consecutive days. The fasting blood glucose of the rats in 72 h after the last injection was detected. The DM model rats were successfully established when fasting blood glucose value reached 16.7 mmol/L or more. After being fed with a high-fat diet for five consecutive weeks, the DM rats were used for the experiments.	Overexpression miR-21 may stimulate nerve regeneration and the recovery of neural function by inhibition of apoptosis via PDCD4 downregulation.
[46]	↓miR-126	NM	Angiogenesis	T2DM In vitro, In vivo, BKS.Cg-m+/+Leprdb/J(db/db-T2DM), bone marrow stromal cells (BMCs), smooth muscle cells (SMCs), mouse brain (ECs), astrocytes cells culture	Ischemic brain tissue and serum present diminished expression of miR-126. EC-Exo contains elevated levels of miR-126 and induces neurorestorative effects in post-stroke DM mice. EC-Exo treatment force angiogenesis, growth of PCN, increased myelin and vascular density. EC-Exo treatment improves cognitive and neurological function.
[36]	↓miR-146a	IRAK1 TRAF6 Ago2	Inflammation	The direct effect of HG on distal axonal growth In vitro, rats DRG neurons, cultured under HG and sildenafil conditions	HG-induced downregulation of miR-146a leads to reduced axonal growth in DRG neurons. Sildenafil treatment reverses the HG-induced effect of axonal growth reduction. Downregulation of miR-146a exacerbates diabetes wound-healing.
[39]	↑miR-181a	ATG5, LC3b-II Bcl-2	Apoptosis, Autophagy,	T1DM STZ-induced T1DM model, In vitro, In vivo, mice, trigeminal ganglion, corneas,	Inhibition of miR-181a upregulates ATG5 and Bcl-2 levels and therefore enhances autophagy and antagonizes apoptosis and promotes neurodegeneration by neuronal axon growth of corneal epithelium in HG condition and therefore play a neuroprotective role in DM mice.
[38]	↑miR-34c	Atg4B LC3-II	Autophagy,	T1DM STZ-induced T1DM model In silico, In vitro, In vivo, mice, trigeminal ganglion, corneas,	MiR-34c inhibition promotes growth of trigeminal ganglion cells. Additionally, miR-34c inhibition restores corneal nerve function via directly targeting both Atg4B and LC3-II. Inhibition of miR-34c accelerates epithelial wound healing of cornea in DM mice by autophagy.
[41]	↑miR-182, ↑miR-96, ↑miR-183, ↑miR-211, ↑miR-204 ↑miR-124 ↓miR-199a-3p, ↓miR-10b, ↓miR-10a, ↓miR-219-2-3p, ↓miR-144 ↓miR-338	NM	Capillary endothelial function	STZ-induced diabetes model *, In vitro, In vivo, rats, retinas *Male Sprague-Dawley rats were fed with standard rat chow and water ad libitum. DM was induced by a single intraperitoneal injection of STZ at a dose of 60 mg/kg body weight, and was defined as a blood glucose level above 16.7 mmol/l determined at 3 days after injection.	The study shows that altered miRNA expression profiles are associated with diabetic retinopathy development. Thus, modulation of those miRNAs may be potentially useful in diabetic retinopathy treatment.
[47]	↑miR-497	BDNF	Apoptosis NEAT1/BDNF/miR-497	STZ-induced diabetes * In silico, In vitro, rats, retinas, primary Muller glial cells * The Male Sprague Dawley rats in the diabetes group received STZ (65 mg/kg) by intravenous injection once. The rat with blood glucose levels>16.7 mmol/L after 5 days of injection was considered successful. After 4 weeks of induction, all rats were sacrificed to isolate the retinal tissues.	Decreased levels of NEAT1 increased the HG-induced apoptosis ratio of Müller cells via regulating miR-497/BDNF axis and promoted retinopathy progression. Injections of pcDNA-NEAT1 enhanced retinal NEAT1 expression and decelerated retina thickening in DM rats.

↑ indicates upregulation, ↓ indicates downregulation of the miRNAs. Abbreviations: Akt, Protein kinase B; Ang-1, Angiopoietin 1; Atg4B, Autophagy related gene 4B; Atg5, Autophagy related gene 5; Bax, BCL2 Associated X Protein; Bcl-2, B-cell lymphoma 2; BDNF, Brain-Derived Neurotrophic Factor; CSN, Central nervous system; CSVD, Cerebral small vessel disease; Cyt-c, cytochrome c; DRG, Dorsal root ganglia; EC-Exo, exosomes derived from mouse brain endothelial cells; FasL, Fast Ligand; GAD-Ab, Glutamic Acid Decarboxylase Antibodies; GFAP, Glial Fibrillary Acidic Protein; GSK3, Glycogen synthase kinase 3; HG, high glucose; HNA, Hereditary neuralgic amyotrophy; HO-1, Heme oxygenase-1; IEFN intraepidermal nerve fibers; IRAK1, Interleukin 1 Receptor Associated Kinase 1; LC3-II, Microtubule-associated protein light chain 3 II; MIAT, Myocardial Infarction Associated Transcript; NF-kB, Nuclear Factor kappa B; NGF, Nerve Growth Factor; NM, not mentioned; NOX4, NADPH oxidase 4; NEAT1, Nuclear Paraspeckle Assembly Transcript 1; Nrf2, Nuclear factor erythroid 2-related factor 2; NT-3, Neurotrophin-3; PCNs, primary cortical neurons; PDCD4, Programmed Cell Death Protein 4; PDE3A, Phosphodiesterase 3A; PTEN, Phosphatase and Tensin Homolog; Sirt1, Sirtuin 1; STZ, streptozocin; TLR, Toll-like Receptor; TRPM7, Transient receptor potential cation channel, subfamily M, member 7; TRAF6, TNF Receptor Associated Factor 6; UN, unaltered; VEGF, Vascular Endothelial Growth Factor; T1DM, type 1 diabetes mellitus.

**Table 2:** Potential lncRNAs involved in neurodegeneration.

Ref	Analyzed lncRNAs and Their Deregulation	Analyzed mRNAs	Pathophysiological Mechanism and Axis	Methodology	Conclusion
[74]	↑PVT1	PI3K/ AKT	Apoptosis, PI3K/AKT signaling pathway	STZ-induced diabetes * In vitro, In vivo, rats, DRG * Male Sprague Dawley rats were injected 55 mg/kg of STZ intraperitoneally. The diabetes rat model was considered to be successfully established when the fasting blood-glucose level in rats was detected to be higher than 16.67 mmol/L.	PVT1 can significantly inhibit the apoptosis of DRG cells in diabetic rats. PVT1 protects DPN via inhibiting the PI3K/AKT pathway
[19]	↓MIAT	VEGF, BDNF, NGF, NT-3, Ang-1	Angiogenesis, MIAT/miR-150-5p/VEGF axis	STZ-induced diabetes *, In vivo, In vitro, mice, corneas, retinas * Study used single injection of STZ to mice, 60 mg/kg used to induce diabetes. Seven days after STZ injection, animals with blood glucose levels > 16.7 mmol/L were included in diabetic group.	MIAT regulates neural and vascular cell function via MIAT/miR-150-5p/VEGF network. MIAT knockdown leads to microvascular degeneration, progressive neuronal loss, neurodegeneration and behavioral deficits in a CNS. MIAT may represent a pharmacological target for treating neurovascular-related disorders.
[47]	↓NEAT1	BDNF	Apoptosis NEAT1/BDNF/miR-497	STZ-induced diabetes * In silico, In vitro, rats, retinas, primary Muller glial cells * The Male Sprague Dawley rats in the diabetes group received STZ (65 mg/kg) by intravenous injection once. The rat with blood glucose levels>16.7 mmol/L after 5 days of injection was considered successful. After 4 weeks of induction, all rats were sacrificed to isolate the retinal tissues.	Decreased levels of NEAT1 increased the HG-induced apoptosis ratio of Müller cells via regulating miR-497/BDNF axis and promoted retinopathy progression. Injections of pcDNA-NEAT1 enhanced retinal NEAT1 expression and decelerated retina thickening in DM rats.
[75]	↑RNCR3	GFAP Vimentin	Apoptosis, Inflammation,	STZ-induced diabetes * In vitro, In vivo, mice, primary Muller glial cells, retinas * Male C57BL/6 mice (4-month old) were used for the induction of diabetes. Diabetes was induced by the intraperitoneal injection of STZ (70 mg/kg). Blood glucose level was measured, in non-fasted animals, in venous blood using a glucometer. Blood glucose level ≥16.7 mmol/L was considered to be hyperglycemia	RNCR3 knockdown treatment reduces IL-2, IL-3, IL-4, IL-5, IL-9, IL-13, IL-17, MCP-1, VEGF and TNF-α. RNCR 3 knockdown could inhibit retinal glial reactivity and prevent HG-induced retinal neurodegeneration and may be used as a treatment.
[76]	↑H19	Bax, Caspase-3, IGF2, Bcl-2, CAT, SOD2, GPx4	Apoptosis, Oxidative stress,	STZ-induced diabetes * In vitro, In vivo, mice, hippocampal cells * Male 6 weeks old Kunming mice were injected intraperitoneally with STZ (70 mg/kg) to induce diabetes. After the STZ injections, blood from the tail vein was collected accordingly. The mice that were confirmed to have a blood glucose concentration greater than 18 mmol/L were deemed to have been successfully modeled.	Downregulation of H19 results with downregulation of Bax, Caspase-3 and upregulation of Bcl-2 therefore inhibits apoptosis of hippocampal neurons in DM mice, and could be a potential treatment target. H19 knockdown diminishes oxidative stress by decrease of LPO and enhances GSH, CAT, SOD and GSH-Px.
[77]	↓Sox2OT	NRF2, HO1	Oxidative stress, Apoptosis	STZ-induced diabetes * In vitro, In vivo, mice retinal ganglion cells (RGCs) * Adult male C57BL/6 J mice were used in the study. Diabetes was induced by intraperitoneal injection of STZ (70 mg/kg). Animals with blood glucose levels higher than 16.7 mmol/L were deemed as having diabetes.	Sox2OT knockdown decreases apoptosis ratio via downregulation of Bax, Bxl-2 and Caspase-3. Sox2OT knockdown protects against HG condition RGC damage through the NRF2/HO1 signaling pathway, and may become a therapeutic target of DM-induced neural retinal neurodegeneration.
[78]	↑MALAT1	-	Morphological and functional changes in retinal photoreceptor cells	STZ-induced diabetes *, In vivo, mice * Eight weeks old male C57BL/6 mice with no ocular diseases were used. Except fasting for diabetes establishment, food and water available at all times. Mice were intraperitoneally injected with STZ (50mg/kg) for 5 consecutive days after an 8h fast to induce diabetes. On the 7th day after first injection, blood samples were collected to measure blood glucose concentration. Effective induction of the diabetes was defined as glucose levels greater than 300 mg/dL.	Retinal MALAT1 knockdown presents mitigative effects on rod and cone cells, thus MALAT1 silencing can be potentially used in diabetic neurodegeneration therapy
[79]	↓GAS5	TNF-α, IL-1β, IL-6	Inflammation, Apoptosis, Endoplasmic reticulum stress	In vitro, HG condition, ARPE-19 human adult retinal pigment epithelial cells	The treatment of GAS5 suppressed endoplasmic reticulum stress-induced apoptosis and inflammation in HG-treated cells.

↑ indicates upregulation, ↓ indicates downregulation of the lncRNAs. Abbreviations: Ang-1, Angiopoietin 1; Atg5, Autophagy related gene 5; Atg7, Autophagy related gene 7; Bax, BCL2 Associated X; Bcl2, B-cell lymphoma-2; BDNF, Brain-derived neurotrophic factor; CAT, catalase; CNS, Central nervous system; DPN, Diabetic peripheral neuropathy; DRG, Dorsal root ganglia; GAS5, growth arrest-specific transcript 5; GFAP, Glial fibrillary acidic protein; GSH, glutathione peroxidase; HG, High glucose; IGF2, Insulin like growth factor-2; IL-1β, Interleukin-1β; IL-6, Interleukin-6; LC3I, Microtubule-associated protein light chain 3 I; LC3II, Microtubule-associated protein light chain 3 II; LPO, lipid peroxide; MALAT1, metastasis-associated lung adenocarcinoma transcript 1; MCP-1, Monocyte chemoattractant protein 1; MIAT, Myocardial infarction associated transcript; MSC, Mesenchymal stromal cells; NGF, Nerve growth factor; NRF2/HO1, Nuclear factor erythroid 2-related factor 2/heme oxygenase-1; NT-3, Neurotrophin-3; NEAT1, Nuclear paraspeckle assembly transcript 1; PI3K/AKT, Phosphatidylinositol 3-kinase/protein kinase B; PVT1,Plasmacytoma variant translocation 1; RGC, retinal ganglion cells; RNCR3, Retinal non-coding RNA3; SOD, superoxide dismutase; STZ, streptozocin; TNF-α, Tumor Necrosis Factor α; VEGF, Vascular endothelial growth factor.

## Abbreviations

AGEs	Advanced glycation end products
AgRP	Agouti-related protein
AD	Alzheimer’s disease
Aβ	Amyloid-β
Ang-1	Angiopoietin 1
ARC	Arcuate nucleus of the hypothalamus
Atg4B	Autophagy related gene 4B
Bcl-2	B-cell lymphoma 2
BDNF	Brain-derived neurotrophic factor
CNS	Central nervous system
CSVD	Cerebral small vessel disease
CART	Cocaine and amphetamine-related transcript
DM	Diabetes Mellitus
DCN	Diabetic corneal neuropathy
DPN	Diabetic peripheral neuropathy
Dicer	Endoribonuclease
DIO	Diet-induced obesity
DRG	Dorsal root ganglia
ECs	Endothelial cells
GAS5	Growth arrest-specific transcript 5
GFAP	Glial fibrillary acidic protein
GFP	Green fluorescent protein
HO-1	Heme oxygenase-1
HG	High glucose
hCMEC/D3	Human cerebral endothelial cell model
HD	Huntington’s Disease
IRS-1	Increased insulin receptor substrate 1
iPSC	Induced pluripotent stem cells
IRS-1	Insulin receptor substrate 1
IRAK1	Interleukin 1 Receptor Associated Kinase 1
IL-1β	Interleukin-1β
IL-2	Interleukin 2
IL-6	Interleukin 6
IP	Intraperitoneal
LARP7	La ribonucleoprotein domain family member 7
lncRNA	Long noncoding RNA,
MSC	Mesenchymal stromal cells,
MALAT1	Metastasis-associated lung adenocarcinoma transcript 1
miRs	MicroRNAs, miRNAs
LC3-II	Microtubule-associated protein 1 light chain 3
miRISC	MiRNA induced silencing complex
MCP-1	Monocyte chemotactic protein-1
NOX4	NADPH oxidase 4
NTC	Negative Treated Control
NPY	Neuropeptide Y
NEAT1	Nuclear paraspeckle assembly transcript 1
Nrf2	Nuclear factor erythroid 2-related factor 2
NF-κB	Nuclear factor kappa-light-chain-enhancer of activated B cells
PD	Parkinson’s Disease,
PTIP 1	Pax transactivation domain-interacting protein 1
p-NFkB	Phospho NFkB
PDE3A	Phosphodiesterase 3a
POMC	Pro-opiomelanocortin
PDCD4	Programmed cell death protein 4
Ago-2	Protein argonaute-2
qPCR	Quantitative real-time PCR
ROS	Reactive oxygen species
RAGE	Receptor for advanced glycation end
RGCs	Retinal ganglion cells
Sirt1	Silent mating type information regulation 2 homolog 1
Sox2OT	Sox2 overlapping transcript
STZ	Streptozocin
Tβ4	Thymosin beta 4
TRAF6	TNF Receptor Associated Factor 6
TLR4	Toll-like receptor 4
TRPM7	Transient receptor potential melastatin 7
TRAF6	Tumor necrosis factor (TNFR)-associated factor 6
TNF-α	Tumor necrosis factor α
T1DM	Type 1 Diabetes Mellitus
UTR	30 untranslated region
VCAM-1	Vascular cell adhesion molecule-1
VEGF	Vascular endothelial growth factor
YFP	Yellow fluorescent protein
